# Approved Societies and Hospital Treatment

**Published:** 1922-07

**Authors:** F. Squires


### Approved Societies and Hospital Treatment.

*To the Editor of* THE HOSPITAL AND HEALTH REVIEW.

SIR,—My attention has been drawn to a letter by Sir James Michelli, under the above title, in which he states that the Hearts of Oak Approved Society has disposed of its assets in the form of additional cash benefits to its members. Apparently he is referring to the Hearts of Oak Benefit Society.

I have been assured that the *Hearts of Oak Assurance Co., Ltd.*, of Holborn Viaduct, which is the Approved Society allied to the National Amalgamated Approved Society, is carrying on this scheme as mentioned in the article referred to.

Yours faithfully,
F. SQUIRES.

### Help for Armenia.

*To the Editor of* THE HOSPITAL AND HEALTH REVIEW.

